# Exploring Predictive Risk Factors for Myocardial Injury in Children Treated with Anthracyclines: A Pilot Study

**DOI:** 10.1007/s12012-025-10065-9

**Published:** 2025-10-17

**Authors:** Taewon Lee, David Douglass, Kimo Stine, Bounleut Phanavanh, Nysia George, Vikrant Vijay, James C. Fuscoe, Varsha G. Desai

**Affiliations:** 1 Division of Applied Mathematical Sciences, College of Science and Technology, Korea University, Sejong, Republic of Korea; 2 Department of Pediatrics, Hematology/Oncology Section, University of Arkansas for Medical Sciences, Little Rock, AR USA; 3 Division of Systems Biology, National Center for Toxicological Research, U.S. Food and Drug Administration, Jefferson, AR USA; 4 Division of Bioinformatics and Biostatistics, National Center for Toxicological Research, U.S. Food and Drug Administration, Jefferson, AR USA; 5 Department of Research and Development, Dr. Vishwanath Karad MIT World Peace University, Pune, Maharashtra India

**Keywords:** Anthracyclines, Myocardial injury, Logistic regression, Pediatric cancer, Risk prediction model

## Abstract

Anthracycline-induced cardiotoxicity poses a threat to the long-term heart health of childhood cancer survivors; however, early risk assessment remains challenging due to limited predictive tools. While risk prediction models have been developed for chemotherapy-related hematological toxicity in pediatric cancer patients, research efforts addressing anthracycline-induced cardiotoxicity in this population remain limited. To fill this gap, we conducted a pilot study to develop a risk prediction model for anthracycline-induced cardiotoxicity in children. Using a paired-sample design, we analyzed data collected throughout treatment from 18 children receiving anthracycline-based chemotherapy. Patient demographics, clinical features, and treatment regimen served as input (explanatory) variables, while plasma concentration of high-sensitivity cardiac troponin T was used as the outcome (response), representing anthracycline-induced myocardial injury. Of the initial 33 potential variables, the top 18 were selected based on their importance scores in relation to myocardial injury. This set was further refined to 13 by removing redundancies using the *caret* package in R to develop a preliminary logistic regression model. A Leave-One-Patient-Out Cross-Validation identified four key predictors for the final model: sex, age at diagnosis, total cyclophosphamide dose (mg), and days since the first anthracycline dose. All were significantly associated with myocardial injury. The final logistic regression model achieved an accuracy of 85%, a sensitivity of 80%, a specificity of 88%, an area under the curve of 0.89, and a Youden’s index of 0.68 for predicting myocardial injury risk. These preliminary findings suggest the potential of our predictive model to stratify risk of anthracycline-induced myocardial injury in the pediatric population.

**Graphical Abstract:**

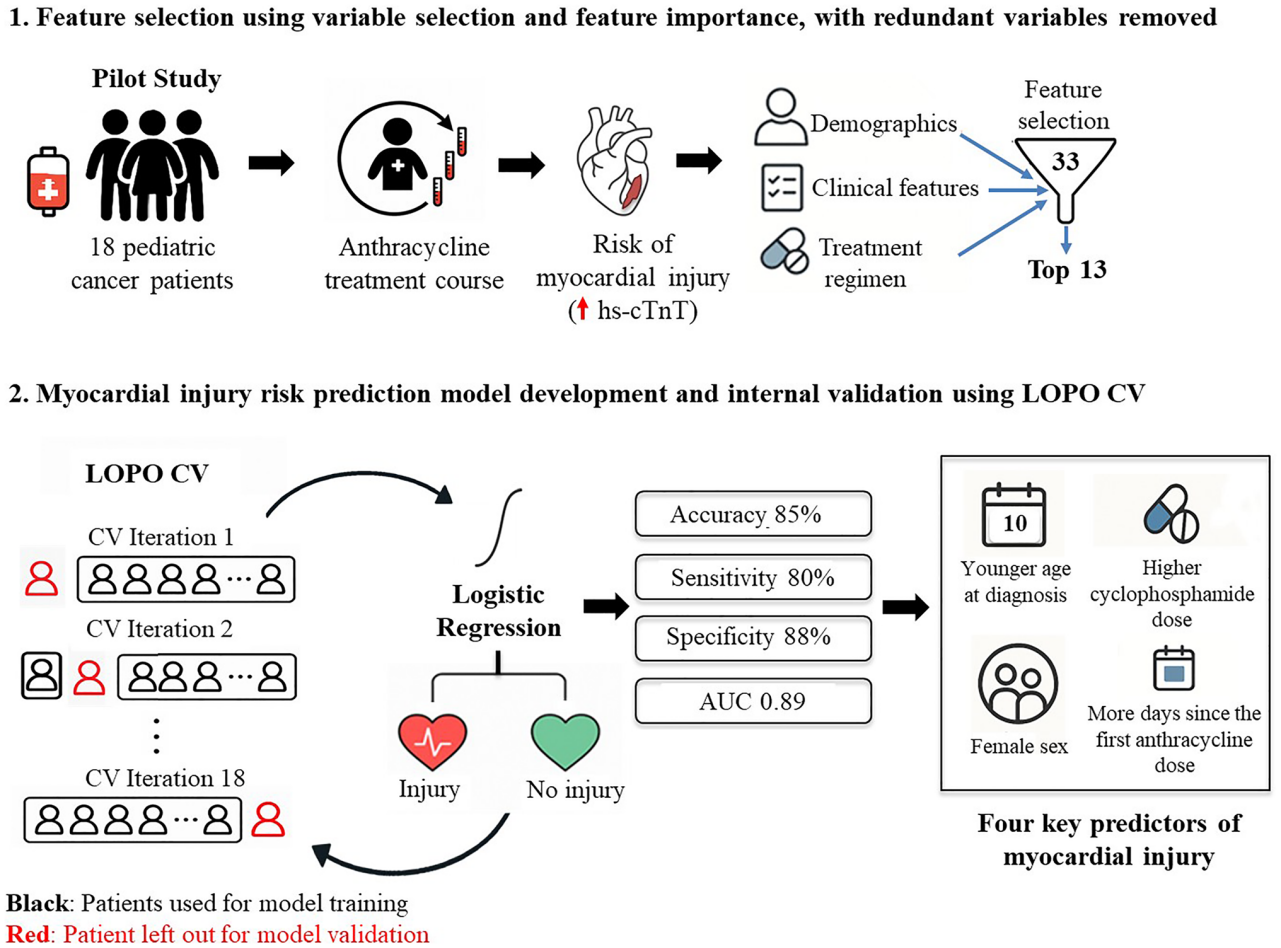

**Supplementary Information:**

The online version contains supplementary material available at 10.1007/s12012-025-10065-9.

## Introduction

Anthracyclines are highly potent anticancer drugs widely used in pediatric oncology to treat a range of childhood cancers, including leukemia and lymphoma [1]. However, these drugs are also known to cause cardiotoxicity, which can manifest as early as within a year of treatment or as delayed heart dysfunction, even years later. Anthracycline-induced cardiotoxicity is a continuous phenomenon that begins with subclinical myocardial injury and progresses through LVEF decline to overt heart failure, representing sequential stages of a single evolving condition rather than three distinct diseases [2]. Potential risk factors such as total cumulative dose, sex, age at treatment, concomitant treatment with other anticancer drugs, and genetic polymorphisms have been proposed as contributing to anthracycline-induced cardiotoxicity [3]. Among these, the total cumulative anthracycline dose is considered a major risk factor in the development of anthracycline-induced cardiotoxicity [4, 5]. In adults who survived childhood or adolescent cancer, a total cumulative anthracycline dose of less than 250 mg/m^2^ doubled the risk of congestive heart failure compared to those who did not receive anthracyclines. This risk increased to 5.2-fold at total cumulative doses of ≥ 250 mg/m^2^, with a higher incidence observed decades after diagnosis [6]. Late cardiotoxic manifestations of anthracyclines have also been reported, even at doses as low as 45 mg/m^2^, in some long-term childhood cancer survivors more than a decade after completing therapy [7]. This implies that there may not be a toxicity threshold at which anthracycline doses can be considered non-cardiotoxic. Moreover, female sex was identified as a vulnerability factor, with female childhood cancer survivors showing a higher risk of congestive heart failure. Female survivors exhibited greater left ventricular dilation [6] and reduced cardiac contractility compared to males [8]. Despite the recognition of several clinical risk factors, accurately identifying children at greater risk for anthracycline-induced cardiotoxicity remains elusive. With a growing population of childhood cancer survivors, there is a critical need for early, precise risk assessment strategies to mitigate the potential for cardiotoxicity in this vulnerable population.

Currently, there is a lack of reliable biomarkers or techniques to predict future heart damage in cancer patients following anthracycline treatment. Routine monitoring of left ventricular systolic function post-anthracycline treatment typically involves 2D echocardiography (ECHO) and multi-gated acquisition (MUGA) scan, which lack sensitivity for the detection of early subclinical cardiotoxicity [3, 9]. While advanced methods like tissue Doppler imaging and speckle tracking have improved myocardial assessment [10], speckle tracking in pediatric survivors remains underexplored [11]. Myocardial strain imaging and cardiac magnetic resonance offer promise but face challenges with reproducibility [12]. In the case of traditional markers (cardiac troponin I and T), although elevated levels are linked to left ventricular dysfunction [13–15], some studies show inconsistent findings. Similarly, N-Terminal pro-Brain Natriuretic Peptide (NT-proBNP) has been linked to an increased risk of cardiac dysfunction, particularly in cases of persistent elevation following chemotherapy [16]. However, its predictive reliability remains uncertain [17]. Despite extensive research, there is no consensus on the most effective approach for predicting post-anthracycline cardiotoxicity.

In recent years, advancements in data science have made risk prediction modeling a critical focus in oncology. Logistic regression models have been developed to identify high-risk breast cancer patients who are susceptible to long-term cardiotoxicity following anthracycline treatment [18–23]. Simultaneously, machine learning-based models have been developed to predict the risk of hematological toxicity associated with various chemotherapeutic agents in pediatric cancer patients [24–26]. However, there remains a significant gap in efforts to model anthracycline-induced cardiotoxicity in pediatric patients with cancer. Only a limited number of studies have developed such risk prediction models incorporating both genetic and clinical risk factors, utilizing statistical approaches such as multiple logistic regression [27, 28] and machine learning algorithms such as random forest [29]. These models, applied to larger cohorts, achieved an area under the curves (AUCs) of 0.77 [27, 28] and 0.72 [29], demonstrating acceptable but suboptimal discrimination between patients who will and will not develop cardiotoxicity. To improve the predictive performance of a cardiotoxicity risk model, we conducted a preliminary study in a small, heterogeneous pediatric cohort, employing an individualized analytical approach to refine risk stratification. In the present study, we focused solely on clinical risk factors, using a logistic regression framework for model development.

## Materials and Methods

### Study Population

This was a prospective pilot study designed to develop a risk prediction model of cardiotoxicity for pediatric cancer patients treated with anthracyclines. Patients were enrolled in the study at Arkansas Children’s Hospital (Little Rock, AR, USA) between July 23, 2018 and December 8, 2021. To determine an appropriate sample size, power analysis was performed using statistical methods designed to control the false discovery rate (FDR) in data analyses. Key assumptions included an FDR of 0.05, a minimum detectable fold-change of 2 in molecular levels between the paired samples, a statistical power of 0.80, and a 95% probability of non-differentially expressed molecular markers, and a common standard deviation. Based on these parameters, the required sample size to detect a twofold change with 80% power was determined to be minimum of 17 patients. When 3 or 4 samples per patient were collected, the minimum detectable odds ratio per 1 standard deviation increase in logistic regression with 80% power was 2.17. Sample size calculation was performed in R using the size package [30].

## Eligibility Criteria

### Inclusion Criteria

To be eligible for the study, participants had to be under 20 years old, weigh at least 2 kg, and could be at any stage of treatment with any prior level of anthracycline exposure. Participants could have received anthracycline infusions at variable durations (ranging from 15 to 30 min pushes to infusions lasting 24 h or more) and were required to have at least one additional planned dose of both an anthracycline and a non-anthracycline agent. Inclusion thresholds also included echocardiographic measurements taken within three months prior to anthracycline administration with M-mode left ventricular ejection fraction (LVEF) ≥ 50% and left ventricular fractional shortening (LVFS) ≥ 28%.

### Exclusion Criteria

The exclusion criteria included any past or present signs of cardiac dysfunction, defined as an LVEF below 50% or an LVFS below 28%. Additionally, individuals with Down syndrome, congenital heart defects, a history of or plans for chest radiation therapy, previous or anticipated high-dose cyclophosphamide exposure (exceeding 2 g/m^2^ per cycle or 1.2 g/m^2^ per dose), or prior or planned treatment with tyrosine kinase inhibitors were not eligible for the study.

### Anthracycline-Based Therapy

The chemotherapy regimens included anthracycline agents (doxorubicin, daunorubicin, and idarubicin) administered concurrently with various combinations of non-anthracycline chemotherapeutic drugs, including cyclophosphamide (CPM), vincristine (VCR), cisplatin (CDDP), tretinoin (ATRA), and/or arsenic trioxide (ATO). Treatment protocols were tailored to each patient's cancer type and conducted in accordance with institutional guidelines.

### Sample Collection

A slightly greater volume of blood, as specified in the consent, was collected as part of routine care. Between three to eight blood samples were collected from each patient during therapy. Plasma samples were sequentially labeled from time points A to H, with a total of 79 samples collected from 18 patients. A paired-sample design was utilized, comparing sequentially collected samples as well as the first and last samples from each patient on anthracycline therapy to evaluate cardiotoxicity outcomes.

This study was conducted in accordance with the U.S. Federal Policy for the Protection of Human Subjects (Common Rule, 45 CFR 46), the principles of the Declaration of Helsinki, and the International Conference on Harmonization Good Clinical Practice guidelines. It was carried out in full compliance with applicable government regulations and the research policies of the University of Arkansas for Medical Sciences (UAMS, Little Rock, AR, USA). It received approval from both the UAMS Institutional Review Board (IRB) and the U.S. Food and Drug Administration’s IRB (Research Involving Human Subjects Committee). Written informed consent was obtained from all study participants, their parents, or legal guardians using an IRB-approved consent form, prior to the initiation of any study-related procedures. All procedures were closely monitored to safeguard the safety of participants, maintain the integrity of the data, and ensure the overall quality of the study. Study samples were assigned unique identifying codes to ensure confidentiality, with samples de-identified to protect patient privacy. The key linking patients’ identities to the codes was securely stored, accessible only to the pediatric hematology oncologists at Arkansas Children’s Hospital (UAMS) involved in the study. Patient demographics, clinical characteristics, and therapy-related data were retrieved from medical records at Arkansas Children’s Hospital, with patient identities redacted.

## Investigations

### Evaluation of Cardiac Function

Cardiac function was assessed by 2D ECHO using a GE Vivid E95 ultrasound system (GE Healthcare, Chicago, IL, USA) in each patient before initiation and during anthracycline therapy at Arkansas Children’s Hospital. Echocardiographic measurements included LVEF and LVFS executed by a trained technician and interpreted by an expert pediatric cardiologist. Alternatively, a MUGA scan was performed if there were concerns regarding the accuracy of the ECHO. A reduction in LVEF below 55% or LVFS below 28% or a consecutive decline of 10% or more in either parameter is indicative of cardiac dysfunction, at which point modifications to further anthracycline therapy are typically considered. Patients were monitored via routine ECHOs, with data recorded only if parameters fell below defined thresholds.

## Assessment of Myocardial Damage

### Processing of Blood Samples

Whole blood was collected in lithium-heparin-coated vacutainer tube from each patient and subsequently centrifuged at 1000×*g* for 10 min at 4 °C to separate the plasma. Plasma samples were stored at − 80 °C until measurements of traditional cardiac disease markers to identify heart damage in patients during anthracycline treatment.

### Plasma Measurements of Traditional Cardiac Disease Markers

The concentrations of cardiac troponin T (cTnT), a marker of myocardial injury, and N-terminal pro-brain natriuretic peptide (NT-proBNP), a marker of cardiac wall stress, were measured in plasma. cTnT levels were measured using the high-sensitivity Elecsys Troponin T Gen 5 STAT immunoassay (Roche Diagnostics, Indianapolis, IN, USA) and are referred to as high-sensitivity cardiac troponin T (hs-cTnT) throughout this manuscript. NT-proBNP concentrations were measured using the Elecsys ProBNP II immunoassay (Roche Diagnostics). Both assays were performed on a Cobas e411 analyzer (Roche Diagnostics) following the manufacturer’s instructions. hs-cTnT levels exceeding 14 ng/L in males and 11 ng/L in females [31] and NT-proBNP concentrations greater than 216 pg/mL [32] were considered indicative of heart damage, based on accepted pediatric normal ranges.

## Development of a Risk Prediction Model of Cardiotoxicity

### Data Organization

Data from 79 samples from 18 patients were systematically organized, with each row representing an individual sample. The dataset included patient demographics (e.g., age, sex), clinical characteristics (e.g., BSA, BMI), and treatment-related details (e.g., type and dosage of anthracycline, days since last administration) and these served as input variables. The plasma hs-cTnT level served as the output variable representing the cardiotoxicity outcome, as only one patient demonstrated altered systolic function. A total of 33 potential input variables, along with the plasma hs-cTnT level as the output variable, were structured across the 34 columns, each labeled accordingly. The hs-cTnT was also included as an input variable at the input time point to assess the cardiotoxicity outcome at the subsequent output time point. Further analysis was conducted independently for each patient to evaluate cardiotoxicity outcomes at specific time points, based on input variables from preceding time points, as detailed later in the manuscript.

### Selection of Input and Output Variables

Potential input variables were derived from patient demographics, clinical characteristics, and treatment regimens, selected based on clinical knowledge and scientific literature identifying these factors as risks for anthracycline-induced cardiotoxicity. Previous studies used changes in LVEF as the output variable in development of cardiotoxicity risk prediction models [21, 22, 29, 33–35]. In the present study, however, all patients maintained normal cardiac function throughout anthracycline treatment, except for one White male patient with sarcoma. This patient showed cardiac dysfunction (LVEF < 55% and LVFS < 28%) 55 days after receiving 300 mg/m^2^ total cumulative doxorubicin dose. However, the patient recovered normal cardiac function after treatment with the angiotensin-converting enzyme inhibitor, Lisinopril. Plasma hs-cTnT level was therefore used as a surrogate marker for cardiotoxicity, serving as the model’s output variable for predicting myocardial injury in the present study. Studies have demonstrated a strong correlation between elevated plasma levels of cardiac troponins and left ventricular dysfunction following anthracycline treatment [17, 36–39]. In the present study, however, the low incidence of left ventricular dysfunction in our cohort precluded meaningful statistical assessment of the association between hs-cTnT and ventricular dysfunction.

### Significance of the Input Variables in Predicting the Risk of Myocardial Injury

The paired-sample strategy was employed for each patient, where potential input variables at time point A (input time point) were compared with the output variable at the subsequent time point B (output time point) to predict myocardial injury. This process was repeated sequentially, comparing input variables at time point B with the output variable at time point C, and so forth, across the entire series of samples (ranging from 3 to 8) collected from each patient. This approach allowed for continuous assessment of myocardial injury risk across multiple time points in each patient. For patients undergoing anthracycline treatment at the time of enrollment, the time elapsed since the initial anthracycline dose was factored into the risk assessment for myocardial injury. Additionally, the duration since the most recent anthracycline administration was incorporated into pairwise comparisons to evaluate the risk of myocardial injury during the study period.

Cardiotoxicity was represented as a binary response variable, indicating the presence or absence of myocardial injury based on the concentration of hs-cTnT in plasma. A concentration above the reference value was coded as ‘1’ (myocardial injury), while a concentration below the reference was coded as ‘0’ (no myocardial injury). Application of the paired-sample design to the total of 79 samples collected from 18 patient resulted in 61 paired samples, of which, 41 cases showed hs-cTnT concentration below the reference value at output time point and 20 cases showed hs-cTnT concentration above the reference value at the output time point (14 ng/L in male and 11 ng/L in female). The impact of each potential input variable on the onset of myocardial injury during anthracycline treatment was assessed, with statistical significance evaluated through *p* value. Categorical variables are described as frequencies, whereas continuous variables are reported as mean and standard deviations. Fisher’s exact test or Pearson’s Chi-square was used to compare categorical variables between 2 groups (patients with or without myocardial injury). For continuous variables that were normally distributed, comparisons were evaluated using Student’s t test. Nonparametric tests (Wilcoxon signed-rank test) were applied for continuous variables that were determined not to follow a normal distribution by Shapiro–Wilk test.

### Model Development Using Logistic Regression

**Fig. 1:**
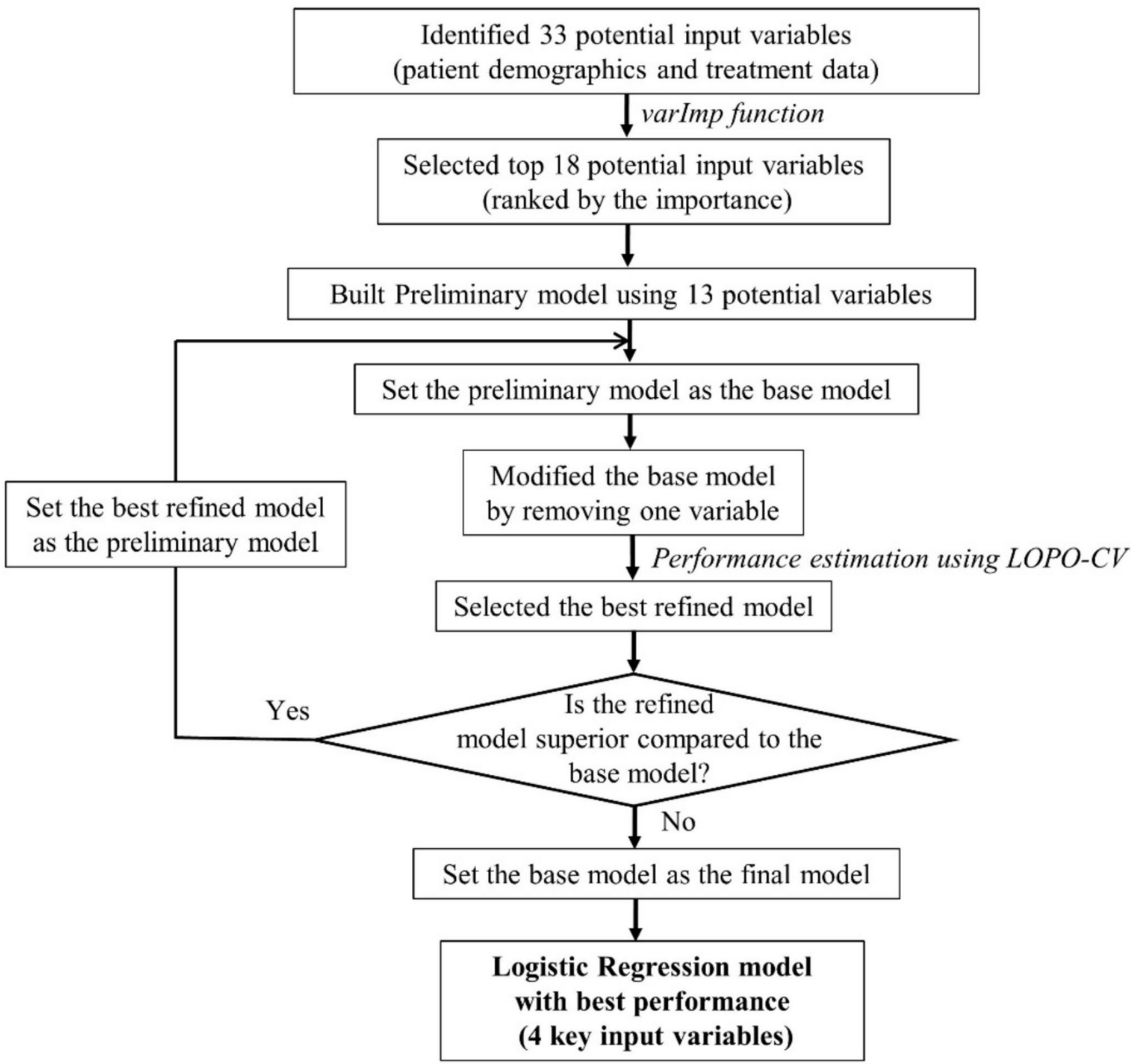
Schematic representation of logistic regression model development

The *caret* (Classification And REgression Training) package in R was utilized for estimating variable importance and model performance through cross-validation method. Figure 1 provides a schematic representation of the systematic approach used to develop a risk prediction model with optimal performance. Additionally, a detailed description of the approach is provided in Supplementary Material S1 and Supplementary Table S1. This process involved four crucial steps: (1) variable selection via variable importance using *varImp* function, (2) performance estimation using Leave-One-Patient-Out Cross-Validation (LOPO-CV), (3) refining the set of the potential input variables, and (4) iterative refinement process to construct the final model with optimal predictive performance. Model performance was evaluated in terms of accuracy, sensitivity, specificity, positive predictive value (PPV), negative predictive value (NPV), an AUC and the Youden’s index [40] using the R package (Statistical Computing, Vienna, Austria).

Thirty-three potential input variables were initially identified from patient demographics, clinical characteristics, and treatment-related data. These variables were ranked by their importance in predicting myocardial injury using the *varImp* function from the *caret* package in R. The top 18 variables were selected, and after removing 5 redundant variables, 13 remained for constructing a preliminary model. This preliminary model served as the base model for further refinement. In each iteration, a variable was removed from the base model to create a refined model, and its performance was evaluated using LOPO-CV method to identify the best-performing refined model. If a refined model outperformed the base model, it was adopted as the new base model for subsequent iterations in the loop. Otherwise, the base model was finalized. This iterative process of refinement, evaluation with LOPO-CV, and model selection continued until an optimal logistic regression model was achieved. The final model, constructed with four key input variables, achieved an accuracy of 85% in predicting the risk of myocardial injury.

### Statistical Significance and Interpretation of the Final Model

The logistic regression coefficient for an independent variable shows the change in the predicted log odds of having an outcome for one unit change of the input variable. The coefficients, *p* values, odds ratios, and 95% confidence interval (CI) of odds ratios were calculated for the final key input variables selected for model development. All analyses were conducted using R Statistical Software (v4.3.1) [41].

### Creation of a Graphical Abstract

Images and icons for the graphical abstract were generated with Microsoft Copilot (copilot.microsoft.com) and subsequently edited using Microsoft PowerPoint and Microsoft Paint.

## Results

### Study Cohort

Nineteen pediatric cancer patients (10 males and 9 females) were initially recruited. However, one female was later deemed ineligible, resulting in a final study cohort of 18 patients (10 males and 8 females). Table 1 summarizes the demographics, clinical characteristics, and treatments of the study population. Among these patients, 11 were newly diagnosed and had not received any chemotherapy at the time of enrollment, while 7 had already started anthracycline-based therapy at the time of enrollment. The cohort included White, African American, and Hispanic patients with a variety of malignancies, such as sarcoma, lymphoma, and leukemia. All patients received anthracyclines concurrently with non-anthracycline agents.

**Table 1:** Demographics and clinical characteristics of the study population

	Male	Female
Age^a^	14.2 ± 4.2 (4 to 19)	10.5 ± 3.9 (5 to 14)
Sex	10 (55.6%)	8 (44.4%)
BSA	1.63 ± 0.48	1.35 ± 0.45
BMI	21.8 ± 7.5	20.4 ± 6.0
*Race*		
White	8 (44.4%)	5 (27.8%)
African American	1 (5.6%)	2 (11.1%)
Hispanic	1 (5.6%)	1 (5.6%)
*Treatment status at enrollment*		
Newly diagnosed	8 (44.4%)	3 (16.7%)
Undergoing therapy	2 (11.1%)	5 (27.8%)
*Type of cancer*		
Sarcoma	7 (38.9%)	5 (27.8%)
Lymphoma	2 (11.1%)	2 (11.1%)
Leukemia	1 (5.6%)	1 (5.6%)
*Anthracycline treatment*^*b*^		
Total cumulative anthracycline dose at the time of enrollment (mg/m^2^)		
0	8 (44.4%)	3 (16.7%)
75	1 (5.6%)	3 (16.7%)
125	0 (0.0%)	1 (5.6%)
150	0 (0.0%)	1 (5.6%)
175	1 (5.6%)	0 (0.0%)
Total cumulative anthracycline dose (doxorubicin isotoxic equivalent dose (mg/m^2^))^c^	183.5 ± 112.9 (75 to 375)	218.1 ± 150.3 (75 to 450)
Total cumulative anthracycline dose < 250 mg/m^2^	126.4 ± 74.4, 7 (38.9%)	140.8 ± 54.4, 6 (33.3%)
Total cumulative anthracycline dose > 250 mg/m^2^	316.7 ± 52.0, 3 (16.7%)	450 ± 0.0, 2 (11.1%)
Days since the first anthracycline dose^d^	169.7 ± 229.7 (28 to 757)	159.2 ± 132.6 (26 to 364)
*Use of cardioprotective drugs*		
Dexrazoxane	3 (16.7%)	3 (16.7%)
Lisinopril	1 (5.6%)	0 (0%)
*Non-anthracycline anticancer drugs*		
Vincristine	8 (44.4%)	6 (33.3%)
Cyclophosphamide	7 (38.9%)	4 (22.2%)
Cisplatin	1 (5.6%)	2 (11.1%)
Arsenic trioxide	1 (5.6%)	0 (0%)
Tretinoin	1 (5.6%)	0 (0%)
*Patients with elevated cardiac disease markers in plasma during treatment*^*e*^		
hs-cTnT	2 (11.1%)	7 (38.9%)
NT-proBNP	2 (11.1%)	3 (16.7%)
*Concentration of cardiac disease markers in plasma during treatment*^*f*^		
hs-cTnT level (ng/L)		
Normal	8.4 ± 2.4, 38 (86.4%)	8.2 ± 2.1, 16 (45.7%)
Abnormal	20.6 ± 4.5, 6 (13.6%)	22.4 ± 13.6, 19 (54.3%)
NT-proBNP (pg/mL)		
Normal	52.4 ± 41.1, 41 (93.2%)	75.4 ± 61.7, 31 (88.6%)
Abnormal	487.4 ± 426.5, 3 (6.8%)	443.3 ± 252.0, 4 (11.4%)

^a^Age in years at diagnosis. The age range is given in parentheses. The median age was 15 years for males and 12 years for females. ^b^Included doxorubicin, daunorubicin, and idarubicin. Total cumulative doses of daunorubicin and idarubicin are expressed as doxorubicin isotoxic equivalent dose in mg/m^2^. ^c^The total cumulative anthracycline dose at the final blood sample collected from the patients while on the study. Range in mg/m^2^ is given in parentheses. ^d^The number of days between the administration of the first anthracycline dose and the last sample collection in the study. The range of days on the study is given in parentheses. ^e^Plasma concentrations of hs-cTnT greater than the reference value (14 ng/L in males and 11 ng/L in females) and NT-proBNP greater than the reference value (216 pg/mL) in at least one of the samples collected from cancer patients during anthracycline treatment, indicating myocardial damage. ^f^Forty-four blood samples from 10 male patients and thirty-five blood samples from 8 female patients were collected during anthracycline treatment. Values are presented as mean ± standard deviation or N (% of the cohort or % of total number of samples collected from male and female patients). *BMI* body mass index (kg/m^2^); *BSA* body surface area (m^2^); *hs-cTnT* high-sensitive cardiac troponin T; *NT-proBNP* N-terminal pro-brain natriuretic peptide

### Patient Demographics

The demographics and clinical characteristics of 18 pediatric cancer patients are summarized in Table 1. At diagnosis, patient ages ranged from 4 to 19 years, with a mean age of 14.2 ± 4.2 years for males and 10.5 ± 3.9 years for females. The average body surface area was 1.63 ± 0.48 m^2^ in males, 20.7% higher than in females (1.35 ± 0.45 m^2^). Similarly, the average body mass index was 6.9% higher in males (21.8 ± 7.5 kg/m^2^) compared to females (20.4 ± 6.0 kg/m^2^). Regarding race and ethnicity, most patients were White (72.2%; 8 males, 5 females), followed by African American (16.7%; 1 male, 2 females) and Hispanic (11.1%; 1 male, 1 female). At enrollment, 11 patients (61.1%; 8 males, 3 females) were newly diagnosed, while 7 (38.9%; 2 males, 5 females) were undergoing treatment. The cohort included a diverse range of cancer types: 66.7% had sarcoma (7 males, 5 females), 22.2% had lymphoma (2 males, 2 females), and 11.1% had leukemia (1 male, 1 female). There were no patients with known underlying comorbidities relevant to cardiotoxicity.

### Anthracycline Treatment Regimen

All patients received anthracycline-based chemotherapy that included doxorubicin, daunorubicin, or idarubicin. Total cumulative doses of daunorubicin and idarubicin were converted to doxorubicin isotoxic equivalents following the Children’s Oncology Group Long-Term Follow-Up guidelines [42]. The cumulative anthracycline dose ranged from 75 to 375 mg/m^2^ in males (183.5 ± 112.9 mg/m^2^) and from 75 to 450 mg/m^2^ in females (218.1 ± 150.3 mg/m^2^) (Table 1). Thirteen patients (72.2%; 7 males, 6 females) received a cumulative anthracycline dose below 250 mg/m^2^, while five patients (27.8%; 3 males, 2 females) exceeded this threshold. Among these five patients, four were treated for sarcoma (two males and two females), and one male was treated for lymphoma. The four sarcoma patients received dexrazoxane as a cardioprotective agent before doxorubicin administration. Additionally, two sarcoma patients (one male, one female) received dexrazoxane despite having a total cumulative anthracycline dose below 250 mg/m^2^.

Between three and eight blood samples were collected from each patient during anthracycline treatment. The number of days varied between sample collection time points among the 18 cancer patients. Additionally, the days between the first anthracycline dose and the final blood sample collection ranged from 28 to 757 days (24.9 months) in males and from 26 to 364 days (12 months) in females, with average durations of 169.7 ± 229.7 days for males and 159.2 ± 132.6 days for females. All patients also received concurrent non-anthracycline chemotherapy agents, including various combinations of VCR, CPM, CDDP, ATO, and/or ATRA. The doses and duration of non-anthracycline treatments varied among the 18 patients. The treatment regimens for the 18 study participants are presented in Table 2.

**Table 2:** Potential variables used for model development

Input variables	Output variable—hs-cTnT^a^		*p* value
	0	1	
	(*N* = 41)^b,c^	(*N* = 20)^b,c^	
*Sex*			0.002^e^
Female	12 (29.3%)	15 (75.0%)	
Male	29 (70.7%)	5 (25.0%)	
Age at diagnosis	12.7 ± 4.6	10.1 ± 5.3	0.048^e^
*Race*			0.110
White	32 (78.0%)	14 (70.0%)	
African American	6 (14.6%)	1 (5.0%)	
Hispanic	3 (7.3%)	5 (25.0%)	
*Blood type*			0.134
A negative	0 (0.0%)	2 (10.0%)	
A positive	16 (39.0%)	8 (40.0%)	
B positive	4 (9.8%)	1 (5.0%)	
O positive	16 (39.0%)	9 (45.0%)	
Not reported	5 (12.2%)	0 (0.0%)	
*Type of malignancy*			0.037^e^
Acute promyelocytic leukemia	2 (4.9%)	0 (0.0%)	
Ewing's sarcoma	23 (56.1%)	6 (30.0%)	
Non-Hodgkin Lymphoma-Diffuse Large B-Cell Lymphoma	2 (4.9%)	0 (0.0%)	
Non-Hodgkin Lymphoma—Burkitt's	2 (4.9%)	1 (5.0%)	
Non-Hodgkin Lymphoma—Relapsed—T-LL	0 (0.0%)	3 (15.0%)	
Osteosarcoma	8 (19.5%)	7 (35.0%)	
Rhabdoid tumor (soft tissue sarcoma)	2 (4.9%)	0 (0.0%)	
T-lymphoblastic lymphoma	2 (4.9%)	1 (5.0%)	
Very High-Risk Pre B-Cell Acute Lymphoblastic Leukemia	0 (0.0%)	2 (10.0%)	
NT-pro BNP	70.1 ± 79.6	144.4 ± 209.5	0.140
hs-cTnT	10.2 ± 6.9	16.6 ± 12.9	0.048^e^
Body Surface Area (m^2^)	1.7 ± 0.5	1.3 ± 0.4	0.002^e^
Body Mass Index (kg/m^2^)	22.9 ± 7.0	18.3 ± 4.4	0.003^e^
*Recommended anthracycline daily dose (mg/m*^*2*^*)*			0.745
0	18 (43.9%)	11 (55.0%)	
12	1 (2.4%)	0 (0.0%)	
25	2 (4.9%)	2 (10.0%)	
37.5	17 (41.5%)	6 (30.0%)	
60	3 (7.3%)	1 (5.0%)	
*Total anthracycline dose (mg) at the input time point*			0.697
0	18 (43.9%)	11 (55.0%)	
1 to 50	1 (2.4%)	1 (5.0%)	
51 to 100	17 (41.5%)	8 (40.0%)	
101 to 150	3 (7.3%)	0 (0.0%)	
151 to 200	0 (0.0%)	0 (0.0%)	
201 to 240	2 (4.9%)	0 (0.0%)	
*Number of days of anthracycline treatment between input and output time points*			0.834
0	18 (43.9%)	11 (55.0%)	
1	4 (9.8%)	1 (5.0%)	
2	13 (31.7%)	7 (35.0%)	
3	1 (2.4%)	0 (0.0%)	
4	4 (9.8%)	1 (5.0%)	
6	1 (2.4%)	0 (0.0%)	
*Anthracycline regimen at the input time point*			0.338
Doxorubicin	9 (22.0%)	5 (25.0%)	
Doxorubicin—continuous	3 (7.3%)	3 (15.0%)	
Dexrazoxane + Doxorubicin	9 (22.0%)	1 (5.0%)	
Dexrazoxane + Doxorubicin—continuous	2 (4.9%)	0 (0.0%)	
None^d^	18 (43.9%)	11 (55.0%)	
*Anthracycline regimen until the input time point*			0.005^e^
Doxorubicin	8 (19.5%)	10 (50.0%)	
Doxorubicin—continuous	7 (17.1%)	7 (35.0%)	
Dexrazoxane + Doxorubicin	17 (41.5%)	2 (10.0%)	
None^d^	9 (22.0%)	1 (5.0%)	
*Total cumulative anthracycline dose (mg/m*^*2*^*)*	116.0 ± 109.4	165.0 ± 81.7	0.081
Number of days between input and output time points	34.6 ± 41.1	23.8 ± 12.0	0.126
Number of days since the last anthracycline dose	20.8 ± 20.3	49.8 ± 98.2	0.207
Number of days since the first anthracycline dose	51.6 ± 64.9	191.4 ± 235.4	0.017^e^
*Recommended VCR daily dose (mg/m*^*2*^*)*			0.017^e^
0	24 (58.5%)	9 (45.0%)	
1.5	4 (9.8%)	8 (40.0%)	
2	13 (31.7%)	3 (15.0%)	
*Total VCR dose (mg) at the input time point*			0.151
0	25 (61.0%)	9 (45.0%)	
0.1 to 3	12 (29.3%)	7 (35.0%)	
3.1 to 6	4 (9.8%)	1 (5.0%)	
6.1 to 9.0	0 (0.0%)	1 (5.0%)	
9.1 to 12	0 (0.0%)	1 (5.0%)	
12.1 to 14.0	0 (0.0%)	1 (5.0%)	
*Number of days of VCR treatment between input and output time points*			0.261
0	24 (58.5%)	9 (45.0%)	
1	14 (34.1%)	6 (30.0%)	
2	2 (4.9%)	2 (10.0%)	
3	1 (2.4%)	0 (0.0%)	
4	0 (0.0%)	1 (5.0%)	
5	0 (0.0%)	1 (5.0%)	
7	0 (0.0%)	1 (5.0%)	
*Recommended CPM daily dose (mg/m*^*2*^*)*			0.943
0	26 (63.4%)	13 (65.0%)	
250	3 (7.3%)	1 (5.0%)	
1200	12 (29.3%)	6 (30.0%)	
*Total CPM dose (mg) at the input time point*			0.826
0	26 (63.4%)	13 (65.0%)	
1 to 1000	2 (4.9%)	2 (10.0%)	
1001 to 2000	7 (17.1%)	4 (20.0%)	
2001 to 3000	5 (12.2%)	1 (5.0%)	
3001 to 4000	0 (0.0%)	0 (0.0%)	
4001 to 4824	1 (2.4%)	0 (0.0%)	
*Number of days of CPM treatment between input and output time points*			0.991
0	26 (63.4%)	13 (65.0%)	
1	13 (31.7%)	6 (30.0%)	
2	2 (4.9%)	1 (5.0%)	
*Recommended CDDP daily dose (mg/m*^*2*^*)*			0.861
0	37 (90.2%)	17 (85.0%)	
60	4 (9.8%)	3 (15.0%)	
*Total CDDP dose (mg) at the input time point*			0.228
0	37 (90.2%)	17 (85.0%)	
50	0 (0.0%)	1 (5.0%)	
51	0 (0.0%)	1 (5.0%)	
52	0 (0.0%)	1 (5.0%)	
110	2 (4.9%)	0 (0.0%)	
114	1 (2.4%)	0 (0.0%)	
119	1 (2.4%)	0 (0.0%)	
*Number of days of CDDP treatment between input and output time points*			0.861
0	37 (90.2%)	17 (85.0%)	
2	4 (9.8%)	3 (15.0%)	
*Recommended ATRA daily dose (mg/m*^*2*^*)*			1
0	40 (97.6%)	20 (100.0%)	
12.5	1 (2.4%)	0 (0.0%)	
*Total ATRA dose (mg) administered at the input time point*			1
0	40 (97.6%)	20 (100.0%)	
240	1 (2.4%)	0 (0.0%)	
*Number of days of ATRA treatment between input and output time points*			1
0	40 (97.6%)	20 (100.0%)	
4	1 (2.4%)	0 (0.0%)	
*Recommended ATO daily dose (mg/kg)*			1
0	40 (97.6%)	20 (100.0%)	
0.15	1 (2.4%)	0 (0.0%)	
*Total ATO dose (mg) at input time point*			1
0	40 (97.6%)	20 (100.0%)	
62	1 (2.4%)	0 (0.0%)	
*Number of days of ATO treatment between input and output time points*			1
0	40 (97.6%)	20 (100.0%)	
4	1 (2.4%)	0 (0.0%)	

^a^Cardiotoxicity outcome was assessed by the concentration of myocardial injury marker, hs-cTnT in plasma samples. It is expressed as a binary variable, where the presence of myocardial injury was indicated by ‘1,’ and its absence by ‘0.’ ^b^Application of the paired-sample design to the total of 79 samples collected from 18 patients resulted in 61 paired samples, with 41 cases with hs-cTnT concentration below the reference value and 20 cases with hs-cTnT concentration above the reference value (14 ng/L in males and 11 ng/L in females). ^c^Values are presented as mean ± standard deviation or *N* (% of total number of samples with or without evidence of myocardial injury). ^d^No anthracycline treatment. Non-anthracycline chemotherapy drugs were administered concurrently with anthracyclines. ^e^Statistically significant association of input variables with myocardial injury. Input and output time points represent the time points at which the input variables (patient demographics, clinical characteristics, and treatment regimens) and the output variable (plasma hs-cTnT concentration), respectively, were integrated into the model. *ATO* arsenic trioxide, *ATRA* Tretinoin, *CDDP* cisplatin, *CPM* cyclophosphamide, *hs-cTnT* high-sensitivity cardiac troponin T, *NT-proBNP* N-terminal pro-brain natriuretic peptide, *VCR* vincristine

### Cardiotoxicity Outcomes

Clinically defined cardiotoxicity is characterized by a LVEF of ≤ 55%, according to the guidelines established by the American Society of Echocardiography [43]. In the present study, however, hs-cTnT was selected as the primary outcome instead of a clinical endpoint such as LVEF decline, as only one of 18 pediatric patients showed a measurable reduction in LVEF during therapy, making it impractical to develop and validate a prediction model based on this endpoint. hs-cTnT therefore served as a necessary and biologically meaningful surrogate marker, offering high sensitivity for detecting subclinical myocardial injury before overt changes in left ventricular function become evident, and providing sufficient variability for model development. This is particularly important in pediatric oncology, where early identification of cardiac injury is crucial for enabling timely intervention to mitigate long-term cardiac complications. LVEF decline typically manifests later, sometimes years after anthracycline exposure, whereas hs-cTnT elevations can be detected during or shortly after treatment, allowing earlier risk stratification. Prospective studies have demonstrated that elevations in hs-cTnT during or shortly after anthracycline and trastuzumab therapy predict subsequent declines in LVEF and adverse cardiac outcomes [44, 45]. These findings support the use of hs-cTnT as a clinically useful surrogate endpoint in settings where clinical events such as LVEF decline are infrequent or occur after prolonged follow-up.

While studies have provided evidence linking hs-cTnT levels to clinical outcomes in adult cancer patients, to our knowledge, direct evidence connecting hs-cTnT to endpoints such as LVEF decline or cardiotoxic events in children with cancer is lacking. Only study of children with newly diagnosed acute leukemia (*n* = 39) reported a significantly higher level in plasma hs-cTnT associated with changes in left ventricular myocardial deformation within one week after completion of anthracycline therapy during the induction phase. However, the prognostic relevance of this elevation for subsequent cardiac dysfunction was not evaluated [46].

In the present study, only one patient with sarcoma developed systolic dysfunction, with an LVEF of 53% and an LVFS of 27%, which occurred 55 days after receiving 300 mg/m^2^ total cumulative doxorubicin dose. However, his cardiac function recovered following treatment with Lisinopril. Among the 17 patients with normal cardiac function, plasma hs-cTnT concentrations exceeded the reference value in 9 patients (52.9%; 2 males, 7 females), while NT-proBNP concentrations remained below the reference threshold. Conversely, NT-proBNP levels were elevated above the reference value in 5 patients (29.4%; 2 males, 3 females), whereas hs-cTnT concentrations remained unchanged during anthracycline treatment. Additionally, one male and two female patients exhibited elevations in both cardiac biomarkers above their respective reference values in at least one sample collected during treatment.

A total of 79 blood samples were collected from 18 patients during anthracycline treatment, including 44 samples from 10 males and 35 samples from 8 females. Elevated hs-cTnT concentrations above the reference value were observed in 6 samples (13.6%) from males, with an average of 20.6 ± 4.5 ng/L, and in 19 samples (54.3%) from females, with an average of 22.4 ± 13.6 ng/L (Table 1). These results indicate a higher frequency of myocardial injury in female patients compared to males, despite both groups having relatively equivalent cumulative anthracycline exposures (218.1 ± 150.3 mg/m^2^ vs. 183.5 ± 112.9 mg/m^2^, *p* = 0.584). Similarly, NT-proBNP levels exceeded the reference value in 3 samples from males, with an average: 487.4 ± 426.5 pg/mL and in 4 samples from females, with an average of 443.3 ± 252.0 pg/mL.

### Logistic Regression Model to Predict the Risk of Myocardial Injury

Thirty-three potential input variables ranked based on their importance in predicting myocardial injury, with the top 18 with an importance score exceeding 20% of the top-ranked variable’s score, were retained. Five of these variables were identified as redundant or less relevant and were removed to prevent interference that could reduce accuracy or lead to model overfitting. The remaining 13 input variables were incorporated into a logistic regression model, which was evaluated using LOPO-CV method. This model achieved an accuracy of 79%, a sensitivity of 60%, a specificity of 88%, and an AUC of 0.84. Additionally, the PPV and NPV were 71% and 82%, respectively.

**Fig. 2:**
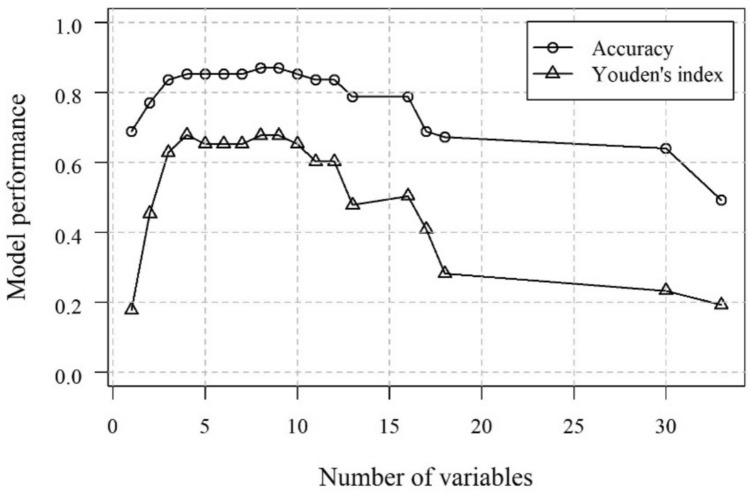
Performance metrics of the best model at different number of variables

To optimize model performance, a stepwise refinement process was employed, iteratively excluding variables and retraining the logistic regression model. Each iteration, assessed via LOPO-CV, continued until no significant improvement was observed. During the LOPO-CV, the model trained on data from 17 patients was used to predict the cardiotoxicity outcome in one patient that was left out. This ensures individualized myocardial injury predictions in patients. Performance was evaluated using predictive accuracy and Youden’s index. A simpler model was favored, when its performance matched that of a more complex counterpart. Figure 2 shows model performance across different variable counts.

The performance of the linear regression model was evaluated using predictive accuracy and Youden’s index, calculated using the leave-one-patient-out cross-validation method. Youden’s index, defined as the sum of sensitivity and specificity minus 1, assesses the effectiveness of a diagnostic test. The X-axis represents the number of variables, while the Y-axis represents the model performance, as indicated by the predictive accuracy and Youden’s index of the best-performing refined model for each variable count. The iterative model refinement process involved removing one variable at a time from the previous best-performing model.

**Fig. 3:**
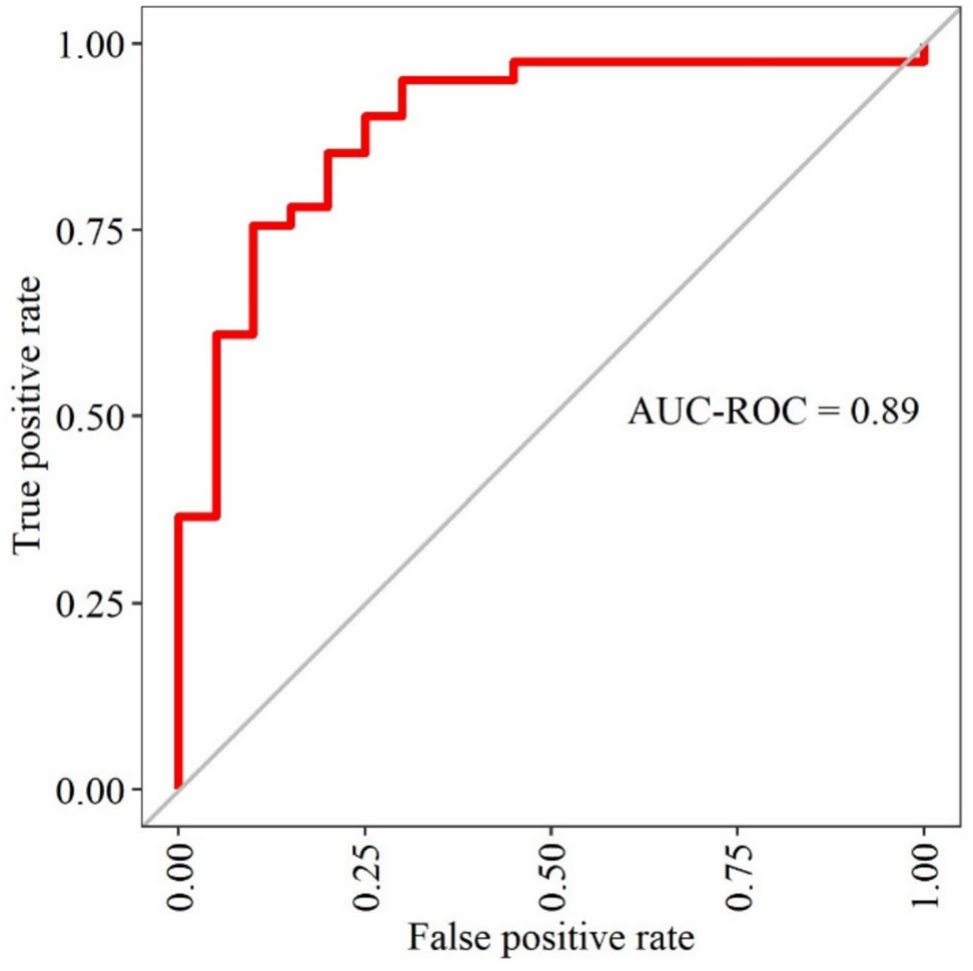
Area under the receiver operating characteristic curve

Ultimately, four key input variables were identified that, when incorporated into the logistic regression model, achieved an accuracy of 85%, a sensitivity of 80%, a specificity of 88%, with Youden’s index of 0.68 and an AUC of 0.89 (Fig. 3). The model also demonstrated a PPV of 76% and a NPV of 90%. These key variables included sex, age at diagnosis, total CPM dose (mg) at the input time point, and the number of days since the first anthracycline dose. Each of these variables demonstrated a statistically significant association in predicting myocardial injury (*p* < 0.05).

The true-positive rate was plotted against the false positive rate using the MLeval and *caret* packages in R. The resulting AUC-ROC was 0.89. AUC-ROC: Area Under the Receiver Operating Characteristic Curve.

### Regression Estimates and Diagnostics of Key Variables

Table 3 presents information on the logistic regression model of the four key input variables with statistical significance of individual regression coefficients (β) tested using the Wald χ2 statistic. These parameters provide insights into the contribution, reliability, and significance of each variable or predictor within the model. These metrics not only estimate the effect size, but also serve as diagnostics for model fit and variable relevance. The regression coefficient (β) represents both the strength and direction of the relationship between each independent variable and the dependent variable in a regression model. In the present study, it describes the effect of predictors on a binary outcome.

**Table 3:** Regression estimates and diagnostics of key variables

Explanatory variable	Regression coefficient (β)	*p* value	OR (95% CI)
Sex	− 4.3994	0.0015	0.0123 (0.0005–0.1203)
Age at diagnosis (yrs)	− 0.2537	0.0242	0.7759 (0.6024–0.9475)
Total CPM dose (mg) at the input time point	0.0012	0.0292	1.0012 (1.0002–1.0025)
Number of days since the first anthracycline dose	0.0160	0.0013	1.0162 (1.0083–1.0292)
Constant	1.0922	0.2973	–
Goodness of Fit (Hosmer and Lemeshow Test)	–	0.4191	–

McFadden’s pseudo-*R*^2^ = 0.5031; *CI* confidence interval; *CPM* cyclophosphamide; *OR* odds ratio

As shown in Table 3, sex affects the likelihood of myocardial injury (*p* < 0.005). Male patients were found to have a reduced probability of experiencing myocardial injury compared to female patients. The negative value of the estimated coefficient (β = − 4.3994, *p* < 0.005) and the odds ratio of less than one (OR = 0.0123) indicate that the probability of myocardial injury is lower in males, with the relative likelihood of decreasing by 98.77% in male patients. Table 3 further reveals that age at diagnosis is a significant predictor of myocardial injury. Older age at diagnosis (*p* < 0.05 and OR = 0.7759) is associated with a significantly lower probability of myocardial injury. Specifically, for each additional year of age at diagnosis, the relative probability of myocardial injury decreases by 22.41%. The total CPM dose (mg) at the input time point was also identified as a significant risk factor for myocardial injury (*p* < 0.05 and OR = 1.0012). With each one mg increase in the total CPM dose, the relative probability of myocardial injury increases by 0.12%. Finally, the number of days since the first anthracycline dose was observed as another significant risk factor (*p* < 0.005 and OR = 1.0162). For each additional day since the first anthracycline dose, the relative probability of myocardial injury increases by 1.62%.

The Hosmer–Lemeshow goodness-of-fit test was also performed in this study. With a *p* value of 0.4191, no significant differences were observed between the actual and expected values of the dependent variable, suggesting that the model provides an adequate fit to the data. A summary of the logistic regression model estimates, including McFadden’s pseudo-*R*^*2*^, is presented in Table 3. The model demonstrates a high McFadden’s pseudo-*R*^2^ value of 0.5031, indicating a strong fit for the logistic regression model with four key variables, as values above 0.4 are generally considered excellent [47].

## Discussion

This study utilized a logistic regression to develop a risk prediction model for anthracycline-induced myocardial injury in pediatric cancer patients. Out of 33 initial potential variables, sex, age at diagnosis, total CPM dose (mg) at the input time point, and the number of days since the first anthracycline dose were identified as four key predictors in a diverse cohort of 18 children and utilized to construct the model. Each predictor demonstrated a statistically significant association with myocardial injury indicated by plasma hs-cTnT levels exceeding the reference value. Evaluation of a 4-variable model using LOPO-CV demonstrated an accuracy of 85%, a sensitivity of 80%, a specificity of 88%, and an AUC of 0.89. Additionally, the PPV and NPV were 76% and 90%, respectively, with a Youden’s index of 0.68. Despite the small sample size, these preliminary results suggest that the model may help identify pediatric patients at increased risk of anthracycline-induced myocardial injury.

### Cardiotoxicity Risk Prediction Models Developed for Pediatric Oncology

In recent years, considerable efforts have been directed toward developing predictive models aimed at facilitating the early identification of at-risk patients and advancing personalized treatment strategies. While the majority of studies have focused on breast cancer patients undergoing anthracycline therapy [18–23], only a couple of studies have explored these models within pediatric cancer populations [28, 29]. Chaix and colleagues evaluated a heterogeneous cohort of 289 childhood cancer survivors, integrating clinical and genetic variables (31 genes enriched for variants) along with LVEF as a cardiotoxicity outcome into a random forest algorithm to predict delayed cardiotoxicity [29]. Their combined clinical-genetic model outperformed single-variable models, achieving sensitivity of 53%, specificity of 91%, and an AUC of 72% compared to 43%, 75%, and 59% for the clinical-only model, and 56%, 87%, and 71% for the genetic-only model. The investigators further emphasized the significance of integrating clinical and genetic predictors to improve the precision of risk stratification for anthracycline-induced cardiotoxicity in pediatric populations.

Visscher et al. (2013) reported similar findings, employing multivariate logistic regression to develop a cardiotoxicity risk prediction model for pediatric cancer patients treated with anthracyclines. An initial model based solely on clinical factors [27] proved suboptimal when applied to a replication cohort comprising patients from Dutch-EKZ and the Canadian Pharmacogenomics Network for Drug Safety [28]. However, the optimized risk prediction model integrating both clinical factors and genetic variants demonstrated significant improvements in predictive performance compared to clinical-only models in distinguishing cases from controls, with AUCs of 0.77 vs. 0.68 in the original cohort and 0.77 vs. 0.69 in the replication cohort. The case patients developed cardiotoxicity during or after anthracycline therapy, while control patients showed no cardiotoxicity despite anthracycline exposure. Key variables included genetic variants and clinical factors including gender and age at treatment initiation, and LVFS as the cardiac outcome [28].

Unlike prior models that combined genetic and clinical data, our logistic regression model was developed exclusively with four clinical predictors—sex, age at diagnosis, total CPM dose (mg) at the input time point, and the number of days since the first anthracycline dose—all of which showed significant (*p* < 0.05) associations with myocardial injury. This 4-variable model presented the most efficient combination of explanatory variables at input time point to predict cardiac injury at the future output time point in the pediatric population studied. The 4-variable logistic regression model exhibited notable discriminatory power (AUC: 0.89), outperforming the logistic regression model with an AUC of 0.77 [28] and the random forest model with an AUC of 0.72 [29], both of which integrated additional genetic factors and employed to larger cohorts. Although the cohort size in our study is relatively small, these preliminary findings suggest that our prediction model may be useful for risk stratification, though this remains subject to further validation.

## Significant Predictors of Myocardial Injury in Pediatric Cancer Patients

### Female Sex

Among patients with myocardial injury, average hs-cTnT levels were higher in females than in males, with elevated levels observed in 54.3% of female plasma samples compared to 13.6% in males, despite relatively equivalent cumulative anthracycline doses (218.1 mg/m^2^ vs. 183.5 mg/m^2^), both are below the conventional threshold of a 250 mg/m^2^ cumulative anthracycline dose, at which the risk of cardiotoxicity rises significantly. Furthermore, analysis of 79 paired samples revealed increased hs-cTnT level in 20 instances, 75% of which were from female cancer patients (*p* = 0.002), underscoring their increased susceptibility to anthracycline-induced myocardial injury. The negative regression coefficient for sex, with females as the reference, also indicated significantly lower risk of myocardial injury in males. Additionally, the odds ratio for males was 0.0123 (95% CI 0.0005, 0.1203), corresponding to an 81.3-fold lower risk compared to females. The narrow confidence interval further supports this association, emphasizing the increased vulnerability of females to myocardial injury. These findings align with prior studies identifying female sex as a major risk factor for cardiotoxic effects in childhood cancer survivors, including higher risks of congestive heart failure [6] and more pronounced cardiac structural and functional impairments [8]. Increased sensitivity in females may stem from physiological differences, such as smaller hearts and distinct cardiac structures [48], potentially affecting cardiac function.

### Younger Age

Studies have shown that children diagnosed before age 10, especially those treated with anthracyclines before age four, are at increased risk for cardiotoxicity [6, 7]. Younger age at diagnosis has been linked to reduced left ventricular mass and thickness as well as elevated left ventricular afterload after receiving anthracyclines [8]. Conversely, patients over the age of 65 are at increased risk for anthracycline-induced heart failure [4], highlighting the critical role of age in influencing cardiotoxicity risk. Consequently, age has been incorporated as a key factor in risk prediction models for pediatric cancer patients [28, 29] and breast cancer patients receiving anthracycline therapy [18–21, 33, 49]. In line with these findings, our study identified younger age as a significant risk factor for anthracycline-induced cardiac injury (*p* = 0.048), with average age in patients with elevated hs-cTnT levels being 10.1 ± 5.3 years compared to 12.7 ± 4.6 years in those without cardiac injury. This is supported by the regression coefficient of − 0.254 (*p* = 0.0242) for age, signifying an inverse association between age and cardiac damage. These results confirm that younger pediatric cancer patients are more vulnerable to anthracycline-induced myocardial injury, which might be partly attributed to developmental and physiological differences, as well as variations in pharmacokinetics.

### Cyclophosphamide Dose

The concurrent administration of anthracyclines with other chemotherapeutic agents, such as CPM, is a recognized risk factor for cardiotoxicity in cancer patients [3, 50, 51]. Studies in adult non-Hodgkin lymphoma patients previously treated with anthracycline-based regimens have demonstrated impaired cardiac function, characterized by reduced LVEF and prolonged corrected QT (QTc) interval and QTc dispersion following high-dose CPM administration [52]. Similarly, acute cardiac alterations such as increased left ventricular diameter and elevated plasma levels of natriuretic peptides have been observed in non-Hodgkin lymphoma patients receiving concomitant anthracycline and CPM therapy [53]. Both anthracyclines and CPM have also been identified as key predictors of cardiotoxicity, leading to their incorporation into machine learning algorithms designed to develop risk prediction models for cardiotoxicity in breast cancer patients treated with anthracyclines [33]. Collectively, these studies underscore the contributory role of CPM in exacerbating anthracycline-induced cardiotoxicity. However, data regarding cardiotoxic events associated with the concurrent administration of anthracyclines and CPM in the pediatric population remain scarce.

In our study, 61% of patients received a concomitant regimen of anthracycline and CPM. Regression analysis revealed a significant positive correlation between total CPM dose (mg) at the input time point and cardiac injury (β = 0.0012), indicating that the odds of cardiotoxicity increased by 0.12% per 1 mg increase in CPM dosage. For instance, a 100 mg increase in CPM would elevate the odds of cardiotoxicity by 12.7%. While sex and age are well-established risk factors, our findings highlight the cumulative cardiac burden associated with anthracycline-CPM coadministration.

### Period After the First Anthracycline Dose

The number of days since the first anthracycline dose is an important predictor of cardiotoxicity risk, particularly when considered alongside other factors. Cardiotoxicity is dose-dependent, with risk increasing over both the cumulative dose and treatment duration. This variable encompasses three key domains: (1) time-dependent risk, where longer exposure increases the likelihood of delayed cardiotoxic effects, (2) cumulative dose effect, where repeated doses elevate the total dose and enhance risk, and (3) early detection, where monitoring in the first months or years may detect subclinical cardiotoxicity before heart failure develops. In the present study, the number of days since the initial anthracycline dose was a significant predictor of cardiac injury in pediatric patients. Patients with cardiac injury had a significantly higher mean duration since the first anthracycline dose (191.4 ± 235.4 days) compared to those without injury (51.6 ± 64.9 days). Also, a positive regression coefficient (β = 0.0160) indicated a positive association between time and risk of myocardial injury, underscoring the importance of time in clinical decision-making and the need for close monitoring as treatment progresses.

### Lack of Established Risk Factors to Predict Myocardial Injury

The total cumulative anthracycline dose (mg/m^2^) was among 33 input (explanatory) variables used during development of a prediction model as it has been a well-established major risk factor of cardiotoxicity. Prior studies have demonstrated that patients receiving doses exceeding 250 mg/m^2^ are at a significantly higher risk of developing congestive heart failure compared to those who did not receive anthracyclines [5, 6]. In our study, however, the average total cumulative doxorubicin equivalent dose was 198.9 mg/m^2^, where 6 out of 18 patients (33%) received dexrazoxane, which may have mitigated the cardiotoxic effects of anthracyclines. Another plausible explanation for the lack of predictive significance of cumulative dose in our model may lie in our small and heterogeneous cohort of 18 patients with varying cancer types and anthracycline treatment (range: 75–450 mg/m^2^).

Additionally, the total cumulative anthracycline dose influences the predictive model through the treatment duration variable, defined as days since the first dose. In other words, patients receiving anthracycline therapy over a longer duration are likely to have received higher cumulative doses. These two variables are moderately correlated (correlation coefficient = 0.5838, *p* < 0.0001) and thus convey similar information for predicting cardiotoxicity. To minimize multicollinearity and potential overfitting, only one of these two variables was retained in the final predictive model. When reducing the model from six to five variables, the predictive accuracy decreased from 85 to 77% upon removing days since the first anthracycline dose, whereas it remained unchanged when cumulative dose of anthracycline was excluded. Therefore, the time variable, days since the first anthracycline dose was selected for inclusion in the final model due to its greater predictive contribution.

Moreover, when both variables were included in the model, the total cumulative anthracycline dose showed an inverse association with the risk of future myocardial injury (Regression Coefficient = –0.0099), which would implausibly suggest that higher cumulative exposure reduces risk. Importantly, the performance metrics (regression coefficients and odds ratios) for the four variables in the current prediction model remained unchanged when days since the first anthracycline dose was retained and cumulative anthracycline dose was excluded.

The schedule of anthracycline administration (i.e., bolus versus continuous) has also been proposed as a potential risk factor for cardiotoxicity. Studies have demonstrated a significantly lower incidence of cardiotoxicity with continuous doxorubicin administration in cancer patients. Hortobagyi et al. (1989) reported that continuous infusion resulted in over 75% fewer heart failure cases at a cumulative dose of 450 mg/m^2^ compared to bolus dosing [54]. Similarly, another study observed cardiotoxicity in 61% of patients receiving bolus administration at a median dose of 420 mg/m^2^, versus 42% with continuous infusion at a higher median dose of 540 mg/m^2^ [55], highlighting the protective effect of continuous infusion despite higher cumulative exposure. In the present study, the anthracycline infusion schedule was included among the 33 input variables in model development. However, it did not emerge as a significant predictor of myocardial injury. This may partly be due to variability in administration protocols (bolus vs. continuous infusion, with or without dexrazoxane) within our cohort of 18 pediatric cancer patients. As a result, infusion schedule was not retained in the final model.

### Study Limitations

The primary limitation of this study is the relatively small cohort of pediatric patients, including diverse cancer types and treatment regimens. Although the power analysis can explain the sample size of 18 patients contributing to 79 blood samples under ideal conditions, observations in the study are not truly independent, but are instead nested within individuals. This data structure increases the risk of overfitting, as the model may inadvertently capture individual-specific patterns or noise rather than generalizable predictors of cardiotoxicity. Although the statistical power is sufficient to detect certain effect sizes under ideal assumptions, the small number of unique individuals in our study limits the model’s external validity and may constrain its applicability to broader clinical populations. Furthermore, logistic regression assumes independence among observations, which may not be fully compatible with the structure of dataset from the present study. Since multiple samples were collected from the same individuals over time, intra-patient correlations are likely present. While LOPO-CV helps mitigate this issue by assessing model performance at the patient level, the modeling framework itself does not directly account for the dependence among repeated measures within individuals. Treating these correlated observations as independent can introduce bias in parameter estimates, underestimate variability, and inflate performance metrics. Therefore, the findings of the present study are interpreted with caution. Future validation in larger, independent cohorts will be crucial to confirm the model’s predictive performance.

The logistic regression model can predict the probability of future cardiotoxicity. In the present study, binary predictions of myocardial injury (hs-cTnT elevation) were derived from these probability estimates to evaluate the accuracy of the predictive model. When the predicted probability exceeds 50%, the outcome is classified as future myocardial injury. A scoring system can be developed based on these probabilities. For example, such a system would generate a specific probability of future myocardial injury for a patient, using their clinical information as input variables. When trained on a larger cohort, the scoring system is expected to produce more stable and reliable probability estimates, making it a valuable tool for personalized anthracycline-based chemotherapy. Therefore, this approach can be applied to larger cohorts in future studies.

Another limitation of this study is the reliance on hs-cTnT as the primary outcome rather than a definitive clinical endpoint such as LVEF decline. Given the limited sample size and relatively short follow-up in the present study, observing sufficient instances of clinically significant LVEF decline was unlikely, whereas hs-cTnT provided a feasible and biologically relevant outcome measure. In our cohort, only one of 18 patients demonstrated a measurable reduction in LVEF during the short follow-up period, making it impractical to develop a predictive model based on this endpoint. Although hs-cTnT served as a surrogate marker that allowed early and sensitive identification of myocardial injury, it cannot fully substitute for clinically meaningful outcomes. Validation in larger cohorts with longer follow-up and incorporation of echocardiographic endpoints will be essential to establish the prognostic relevance of hs-cTnT in predicting ventricular dysfunction.

Finally, we acknowledge that all participants in the present study were recruited from a single center, which may limit the generalizability of our findings. To enhance clinical applicability, external validation in independent cohorts with geographic and demographic diversity is essential. Future research should incorporate larger, multi-center datasets and apply advanced analytical methodologies to improve the robustness, generalizability, and translational potential of these findings.

## Conclusion

This study investigated an innovative personalized logistic regression approach for predicting the risk of myocardial injury in pediatric cancer patients treated with anthracyclines. By utilizing a refined yet clinically relevant set of variables, the model achieved a high AUC of 0.89, reflecting strong ability to discriminate between patients with and without myocardial injury. Furthermore, it demonstrated a predictive accuracy of 85% for future myocardial injury. These preliminary results, supported by favorable performance metrics, provide a basis for validation in larger, independent cohorts with long-term follow-up and may contribute to advancing precision cardio-oncology in pediatric care.

## Supplementary Information

Below is the link to the electronic supplementary material.

Supplementary file1 (DOCX 23 KB)

Supplementary file2 (XLSX 18 KB)

## Data Availability

The data supporting the findings of this study are available from the corresponding author upon reasonable request, in compliance with ethical and privacy regulations.
