# Supplementary material for: Exploring Predictive Risk Factors for Myocardial Injury in Children Treated with Anthracyclines: A Pilot Study
Source: Cardiovasc Toxicol. 2025 Oct 17;25(12):1861–80. doi: 10.1007/s12012-025-10065-9 (PMC13287150; doi:10.1007/s12012-025-10065-9)
Supplement: Supplementary file 1 — Supplementary file1 (DOCX 23 KB) [file 12012_2025_10065_MOESM1_ESM.docx]

Article title: Exploring Predictive Risk Factors for Cardiotoxicity in Children Treated with Anthracyclines: A Pilot Study

Journal name: Cardiovascular Toxicology

Author names: Taewon Lee, David Douglass, Kimo Stine, Bounleut Phanavanh, Nysia George, Vikrant Vijay, James C. Fuscoe, Varsha G. Desai

Corresponding Author: Varsha G. Desai, Ph.D.

Affiliation: Division of Systems Biology, National Center for Toxicological Research, U.S. Food and Drug Administration, Jefferson, Arkansas, USA. (Affiliation at the time the study was conducted)

Present e-mail address: varsha.desai@mitwpu.edu.in

**Supplementary Material S1**: A detailed description of model development

1. **Model development**: The *caret* (**C**lassification **A**nd **RE**gression **T**raining) in package R was utilized for estimating variable importance and model performance through cross-validation method. A systematic approach used for building a logistic regression (LR) model is described below.

**1.1. Variable selection via variable importance**: Important input variables were identified using the *varImp* function within the *caret* package. This function evaluates the contribution of each input variable to the overall predictive power of the model. It provides a ranking of the input variables based on their importance in making predictions. For model development, only highly ranked variables with the importance score of more than 20% of the top-ranked variable's score, were retained. This threshold helped narrow down the most discriminatory variables for the model.

**1.2. Performance estimation using leave-one-patient-out cross-validation**: Accuracy, sensitivity, specificity, positive predictive value (PPV), and negative predictive value (NPV) of the LR model with the important input variables were estimated by a leave-one-patient-out cross-validation (LOPO-CV) method. Leave-one-out cross-validation (LOO-CV) method is primarily used to assess model performance where the model is trained on all data points (observations) except one, and this process is repeated for each data point. The advantage of LOO-CV is that it provides a nearly unbiased estimate of model performance, making it especially valuable for small datasets. In the present study, LOPO-CV approach was chosen over standard LOO-CV or 10-fold CV methods to account for multiple data points within the same patient, which could violate the independence assumption of logistic regression. By leaving out all observations for a single patient at a time, LOPO-CV effectively manages the independence between the input variables and the output variable within individual patients. In the present study, observations from 17 patients were utilized to develop the LR model for predicting cardiotoxicity, with one patient excluded in each iteration. This procedure was systematically repeated for all patients, allowing for the prediction of cardiotoxicity for each individual in turn.

**1.3. Refining the input variable set**: The input variable set of ‘n’ variables was refined by removing a variable that was either not recognized as established risk factors or redundant. The best refined LR model was chosen by the best performing model in the ‘n’ possible modified models with n-1 variables. The performance of a LR model was measured by the predictive accuracy and Youden’s index obtained by LOPO-CV. Youden’s index, the sum of sensitivity and specificity minus 1, is a measure to assess the effectiveness of a diagnostic test. If the new simpler LR model, built with this refined variable set, shows a significant improvement in performance during LOPO-CV, it is deemed a better model for toxicity prediction.

**1.4. Iterative refinement process:** The refinement of the input variable set was repeated iteratively until an optimal set of risk factors was determined or no significant improvement in the model performance was observed. When a new model with a refined set of input variables achieves better performance, it is considered a better model. If the refined model has similar performance to the previous model, the simpler model with fewer input variables is considered better. This iterative approach ensures that the final model is both statistically robust and clinically meaningful, balancing predictive accuracy with the identification of relevant risk factors.

**2. Process to achieve the final refined model**: A step-by-step process to achieve the final refined LR model with four key input variables is described below. The refined input variable set of the best performing model at each step is presented in S1 Table.

**2.1. From 33-varible model to 30-variable model**: Race, blood type, and type of malignancy were removed from input variable set. These 3 variables are categorical variables and may help distinguish between patients, but they are not necessarily predictive of future cardiotoxicity, as the *varImp* function relies on standard logistic regression.

**2.2. From 30-varible model to 18-variable model**: Thirty potential input variables were ranked according to their importance to select the top 18 input variables with an importance score exceeding 20% of the top-ranked variable's score. Consequently, recommended anthracycline daily dose, number of days of anthracycline treatment, anthracycline regimen at the input time point, number of days between input and output time points, number of days of CPM treatment between input and output time points, number of days of CDDP treatment between input and output time points, recommended ATRA daily dose, total ATRA dose (mg) at the input time point, number of days of ATRA treatment between input and output time points, recommended ATO daily dose, total ATO dose (mg) at the input time point, and number of days of ATO treatment between input and output time points were eliminated from input variable set.

**2.3. From 18-variable model to 13-variable model**: Anthracycline regimen until the input time point, which represents anthracycline administration methods (bolus vs. continuous) and dexrazoxane co-administration, can influence cardiotoxicity risk but is secondary to total cumulative anthracycline dose (mg/m²), a key determinant of cardiotoxicity. Including this variable could reduce model accuracy during LOPO-CV, leading to its removal from the set of 18 variables. Additionally, while recommended VCR daily dose and number of days of VCR treatment between the input and output time points provide insight into intended therapy, total VCR dose (mg) at the input time point is a more reliable predictor of cardiotoxicity as it captures actual drug exposure over time. Therefore, recommended VCR daily dose and number of days of VCR treatment between input and output time points were excluded. Similarly, total CPM dose (mg) at the input time point and total CDDP dose (mg) at the input time point were retained in the set, as these are considered to be more relevant to prediction of future cardiotoxicity. In contrast, recommended CPM daily dose and recommended CDDP daily dose were removed from the variable set.

**2.4. From 13-variable model to final 4-variable model**: The iterative refinement process is applied to achieve the final refined model with 4 key input variables. NT-proBNP, hs-cTnT, body surface area, body mass index, total anthracycline dose (mg) at the input time point, total cumulative anthracycline dose (mg/m^2^), number of days since the last anthracycline dose, total VCR dose (mg) at the input time point, and total CDDP dose (mg) at the input time point were sequentially eliminated from the input variable set until the final refined model is achieved.

**2.5. Further refinement from final 4-variable model**: The best refined model with 3 variables was achieved by removing the total CPM dose (mg) at the input time point from the final 4-variable model. However, its performance was comparatively poorer than the final 4-variable model as indicated by an accuracy of 0.84 and Youden’s index 0.63. Further refinement showed a significant drop in model performance (Fig. 2).
